# Prospects for passive immunity to prevent HIV infection

**DOI:** 10.1371/journal.pmed.1002436

**Published:** 2017-11-14

**Authors:** Lynn Morris, Nonhlanhla N. Mkhize

**Affiliations:** 1 National Institute for Communicable Diseases of the National Health Laboratory Service and the University of the Witwatersrand, Johannesburg, South Africa; 2 Center for the AIDS Programme of Research in South Africa (CAPRISA), Durban, South Africa

## Abstract

In a Perspective, Lynn Morris and Nonhlanhla Mkhize discuss the prospects for broadly neutralizing antibodies to be used in preventing HIV infection.

## The need for better HIV prevention tools

Despite the widespread global rollout of antiretroviral therapy and its ability to reduce onward HIV transmission, an alarming 1.8 million new HIV infections are estimated to have occurred in 2016 [1]. Effective methods to prevent HIV infection include condom use and pre-exposure prophylaxis (PrEP) with antiretroviral drugs; however, owing to barriers such as adherence, an effective vaccine would be the most definitive solution to the ongoing burden of HIV infection. Yet researchers have struggled to design a prophylactic vaccine able to induce protective immunity. This highlights the urgency of developing new tools to prevent HIV infections and achieve control of the global epidemic [2]. One such approach is passive immunization with protective antibodies, a strategy that has been used against infectious diseases for over 100 years and has proven useful for post-exposure prophylaxis or against pathogens where no vaccines yet exist [3]. The flourishing field of antibody therapeutics, together with the identification of a growing number of broad and potent HIV monoclonal antibodies, presents an extraordinary opportunity to use this approach for HIV prevention. The protective and therapeutic effects of a number of these broadly neutralizing antibodies (bNAbs) have been well demonstrated in animal studies [4]. Furthermore, bNAbs have been shown to have modest antiviral effects in HIV-infected humans, both in reducing viremia and delaying viral rebound after interruption of antiretroviral treatment [5–7]. What is not yet known is whether bNAbs are able to protect uninfected humans from acquiring HIV.

## Ramping up to AMP

The first bNAb to be tested for efficacy in HIV prevention is VRC01, which was isolated in 2010 following the advent of single-cell technologies that enabled the discovery of a new generation of bNAbs. It targets the CD4 binding site (CD4bs) on the HIV envelope and neutralizes 90% of circulating HIV isolates [8]. VRC01 was shown to be safe and well tolerated in adults in 2 small clinical trials [9,10], as well as in infants in an ongoing phase 1 trial evaluating the potential for preventing mother-to-child transmission [11]. Importantly, VRC01 retains its functional activity following passive infusion with trough levels expected to neutralize the majority of circulating HIV strains [10].

VRC01 is now being tested in 2,700 high-risk homosexual men in the Americas and 1,500 heterosexual women in Africa in 2 separate Antibody Mediated Prevention (AMP) protocols [12]. In these trials, participants receive a total of 10 intravenous infusions of VRC01 once every 8 weeks at a higher (30 mg/kg) or lower (10 mg/kg) dose. Protective efficacy will be assessed by comparing the number of breakthrough HIV infections in the VRC01 groups to a placebo group, while the difference between the 2 dose groups will help to define a protective titer. As a further test of efficacy, viral sensitivity to VRC01 will be assessed in the laboratory. Since natural resistance to VRC01 exists, it will be important to establish whether breakthrough HIV infections are due to preexisting viral resistance or early adaptive escape from VRC01. The AMP proof-of-concept studies have been designed with the explicit purpose of testing whether a bNAb can prevent HIV infection, with results expected by 2020.

## Improved bNAbs in the pipeline

In the past few years, a large number of broader and more potent bNAbs have been isolated, some of which are already in clinical development, including VRC07-523, a CD4bs antibody related to VRC01 [13]. This antibody has been further engineered to include mutations in the Fc region that enhance binding to the neonatal Fc receptor and extend its half-life by up to 6 months [14]. Antibodies targeting other epitopes on the HIV envelope are also being developed for clinical use, including PGT121 and 10–1074 that bind to the high-mannose patch near the V3 region of gp120, as well as the V2 apex antibodies PGDM1400 and CAP256-VRC26.25 that have shown promise in animal studies [15]. Other single antibodies, such as 10E8, that target the membrane proximal external region of gp41, as well as the more recently isolated CD4bs antibody N6, are able to neutralize 98% of global HIV isolates [16]. Bispecific neutralizing antibodies (biNAbs) that are designed to recognize 2 distinct epitopes combine the breadth and potency of the parental antibodies and in some cases have shown synergistic activity [17,18]. Those that target both viral and host cellular proteins, either CD4 or CCR5 viral receptors, have demonstrated dramatically increased potency, probably by pre-positioning the biNAb at the site of viral entry. A recent study of a trispecific antibody (VRC01/PGDM1400/10E8), which combines the breadth and potency of 3 antibodies into one molecule, demonstrated protection in non-human primates against a mixture of SHIVs that were differentially sensitive to the single antibodies [19]. However, while bi- and trispecific antibodies herald new possibilities for improved efficacy, artificial modifications could increase their antigenicity and negatively impact pharmacokinetic properties and safety profiles, but this remains to be tested.

## Challenges of using bNAbs for prevention

Given that no single bNAb can neutralize 100% of viruses, the wisdom of using bNAbs for HIV prevention has been questioned. Added to this is the observation that global viruses exhibit a wide range of sensitivities to individual bNAbs, and neutralization escape from single antibodies can occur readily and rapidly. These concerns could be overcome by combining 3–4 bNAbs that together can cover 100% of viruses [20], although this will substantially increase the cost of the product. The cost of bNAbs is especially important in the era of relatively cheap antiretroviral drugs being used for PrEP to prevent HIV infection. However, drugs have significant side effects, and daily dosing demands high levels of adherence [21] although this concern may be circumvented if long-acting cabotegravir, also being tested on an 8-week dosing schedule, is shown to be efficacious. Nonetheless, antibodies with their excellent safety record and potentially longer half-life could fill a unique niche and increase the options available to those seeking to protect themselves from HIV infection. Furthermore, antibodies possess other functional activities mediated through their Fc regions, such as antibody-dependent cellular cytotoxicity and phagocytosis, that may contribute to protective efficacy [22]. Although the current modality of multiple intravenous infusions has not proven to be a barrier in the clinical experience with VRC01, it is unlikely to be feasible on a population level. Subcutaneous administration could make delivery of bNAbs more practical, but this is only possible with highly potent bNAbs, a property that can also be improved through antibody engineering. Other factors that may undermine the success of bNAbs is our incomplete understanding of mucosal transmission events and the ability of HIV to infect and spread through infected cells or via cell—cell transmission, where bNAbs may less effective [23].

## The future of HIV prevention

The prospect for bNAbs becoming part of the toolbox for reducing new HIV infections will depend on the outcome of the AMP trials and on subsequent testing of broader and more potent bNAbs with longer half-lives as well as lower manufacturing costs. However, even assuming success in ongoing clinical evaluation, it is likely that bNAbs will only be used in selected population groups when they are most at risk, such as young women in Africa or individuals who suspect that they may become exposed to HIV. In this scenario, bNAb administrations every 4–6 months may be a feasible option for a defined time period, akin to the widespread use of medroxyprogesterone acetate for birth control.

Importantly, positive findings from the AMP trials would confirm antibody neutralization as a correlate of protective efficacy, which is critical information as this assumption underlies many HIV envelope immunogen design efforts. In addition, defining antibody levels in the blood and mucosal tissues able to confer protection against HIV infection will also be useful. Of course, this evidence comes with certain caveats because a vaccine would induce multiple antibody specificities, unlike VRC01 that targets a single neutralization-sensitive epitope, and furthermore, active immunization induces innate and adaptive T-cell responses that are not imparted by passive immunization. Nevertheless, minimal protective antibody titers determined in the AMP studies could be used as a benchmark to identify the most promising vaccine candidates at an early stage and thereby accelerate the vaccine discovery process.

**Fig 1:**
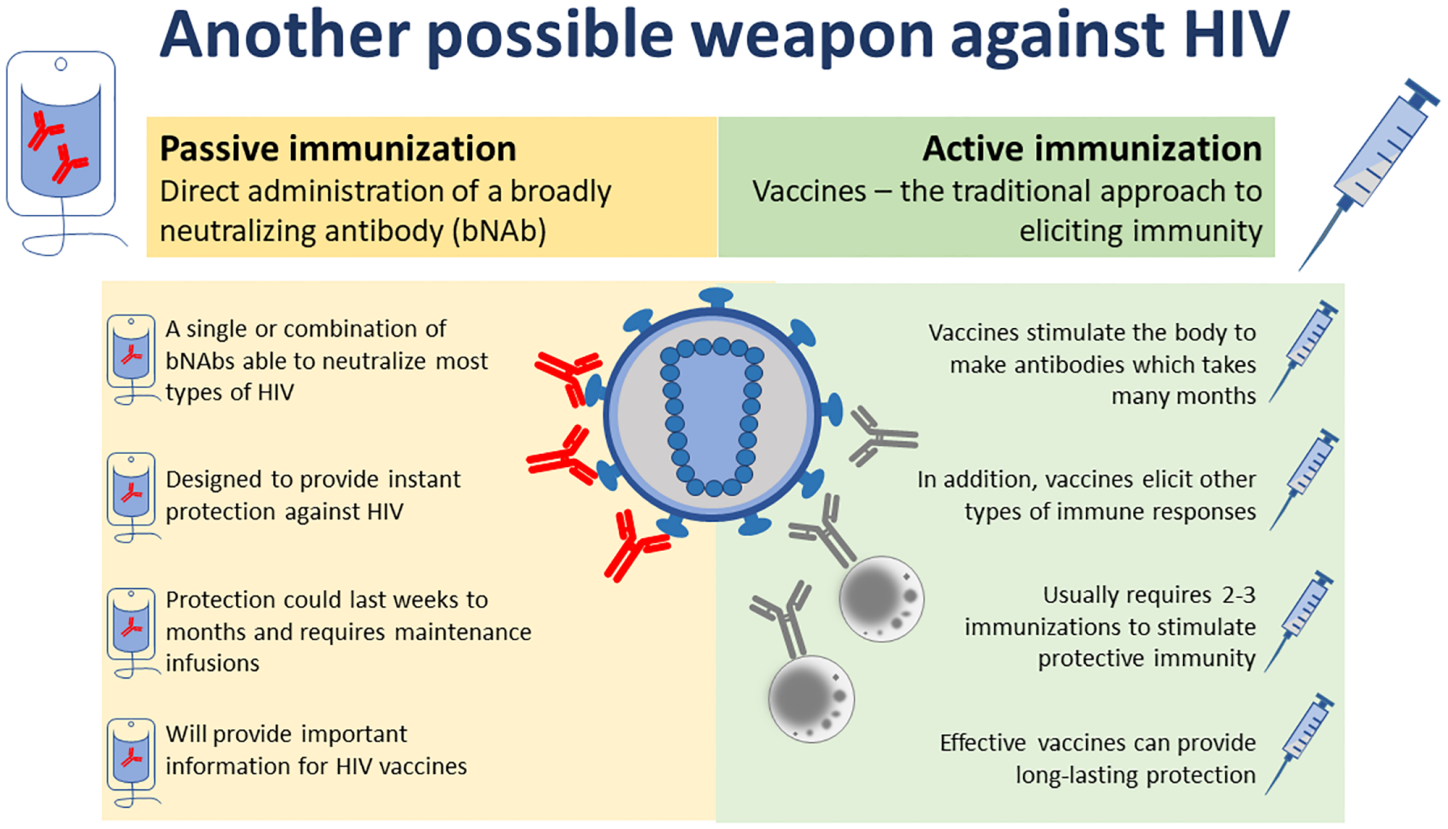
Another possible weapon against HIV. An illustration of the principles of using a monoclonal antibody as passive immunization to prevent HIV infection, as compared with the more traditional vaccine approach of active immunization. *Created by Nolo Moima*, Sunday Times, *and Carina Kriel*, *NICD*.

The pace of progress in development of bNAbs so far, from the discovery of VRC01 to conducting the AMP efficacy trials, has been impressive. Results from this field hold promise not only for a new weapon in the fight against HIV (Fig 1) but also potentially may assist progress in the vaccine field. Contingent on further advances in scientific and clinical studies, the prospects for use of bNAbs as biological drugs for long-term PrEP represent an exciting and innovative approach to tackling the devastating global HIV epidemic. Vector-based delivery is also being explored for sustained production of protective levels of bNAbs [24]. As such, passive immunization could contribute to HIV prevention efforts until a safe and effective HIV vaccine is developed.

## Abbreviations

AMP: Antibody Mediated Protection
biNAbs: bispecific neutralizing antibodies
bNAb: broadly neutralizing antibody
CD4bs: CD4 binding site
PrEP: pre-exposure prophylaxis
